# GPR18 Controls Reconstitution of Mouse Small Intestine Intraepithelial Lymphocytes following Bone Marrow Transplantation

**DOI:** 10.1371/journal.pone.0133854

**Published:** 2015-07-21

**Authors:** Amy M. Becker, Derrick J. Callahan, Justin M. Richner, Jaebok Choi, John F. DiPersio, Michael S. Diamond, Deepta Bhattacharya

**Affiliations:** 1 Department of Pathology and Immunology, Washington University School of Medicine, Saint Louis, Missouri, United States of America; 2 Department of Medicine, Washington University School of Medicine, Saint Louis, Missouri, United States of America; 3 Department of Molecular Microbiology, Washington University School of Medicine, Saint Louis, Missouri, United States of America; 4 Division of Oncology, Washington University School of Medicine, Saint Louis, Missouri, United States of America; Mie University Graduate School of Medicine, JAPAN

## Abstract

Specific G protein coupled receptors (GPRs) regulate the proper positioning, function, and development of immune lineage subsets. Here, we demonstrate that GPR18 regulates the reconstitution of intraepithelial lymphocytes (IELs) of the small intestine following bone marrow transplantation. Through analysis of transcriptional microarray data, we find that GPR18 is highly expressed in IELs, lymphoid progenitors, and mature follicular B cells. To establish the physiological role of this largely uncharacterized GPR, we generated *Gpr18^-/-^* mice. Despite high levels of GPR18 expression in specific hematopoietic progenitors, *Gpr18^-/-^* mice have no defects in lymphopoiesis or myelopoiesis. Moreover, antibody responses following immunization with hapten-protein conjugates or infection with West Nile virus are normal in *Gpr18^-/-^* mice. Steady-state numbers of IELs are also normal in *Gpr18^-/-^* mice. However, competitive bone marrow reconstitution experiments demonstrate that GPR18 is cell-intrinsically required for the optimal restoration of small intestine TCRγδ^+^ and TCRαβ^+^ CD8αα^+^ IELs. In contrast, GPR18 is dispensable for the reconstitution of large intestine IELs. Moreover, *Gpr18^-/-^* bone marrow reconstitutes small intestine IELs similarly to controls in athymic recipients. *Gpr18^-/-^* chimeras show no changes in susceptibility to intestinal insults such as *Citrobacter rodentium* infections or graft versus host disease. These data reveal highly specific requirements for GPR18 in the development and reconstitution of thymus-derived intestinal IEL subsets in the steady-state and after bone marrow transplantation.

## Introduction

The development and function of immune lineages are regulated by cell-extrinsic cues provided by contact with other cells and microbes, the extracellular matrix, and soluble factors. Intestinal IELs, for example, are localized amongst enterocytes and in close proximity to luminal flora such that they can rapidly respond to barrier injury or infection [1]. Intestinal IELs contain several T cell subsets, including conventional CD4^+^ and CD8αβ^+^ TCRαβ^+^ cells and unconventional lymphocytes expressing CD8αα^+^ homodimers [2]. These CD8αα^+^ cells can be further segregated into TCRαβ^+^ and TCRγδ^+^ subsets [2–4]. Each of these subsets likely plays unique functional roles. For example, TCRγδ^+^ IELs limit *Salmonella typhimurium* dissemination following infection and produce keratinocyte growth factor to mediate epithelial regeneration after injury [5–10]. CD8αβ^+^ IELs are particularly important for establishing immunity to certain intestinal pathogens, such as *Toxoplasma gondii*, while CD4^+^ IELs in the large intestine are essential for protection against enteric pathogens such as *Citrobacter rodentium* [11, 12]. Although the functional importance of IELs is increasingly becoming clear, the guidance cues which direct these specialized T cells to colonize the intestinal epithelium are not fully understood.

To ensure access to the appropriate extrinsic signals, IELs must be positioned properly through the combined action of adhesion molecules and chemokine signals [13]. Initial entry into Peyer's patches, the lamina propria, and intraepithelial regions depends upon the expression of integrin β7 [14–16], although the specific α chain pairing depends upon the lymphocyte subset and ultimate destination. Integrin α4β7 mediates binding to the Peyer's Patch high endothelial venules and is essential for entry into the mucosa by conventional CD4^+^ and CD8αβ^+^ lymphocytes [15–17]. Upon activation in secondary lymphoid organs, conventional T cells can also express integrin αE to adhere to E-cadherin-expressing intestinal epithelium [18–20]. In contrast, unconventional CD8αα^+^ IEL precursors express αEβ7 as they exit the thymus and can directly migrate to the intestinal epithelium [21–24]. Integrin β2 is also important for conventional TCRαβ^+^ and activated TCRγδ^+^ cell numbers in the mucosa [25]. Aside from these adhesion molecules, chemokines direct IELs to specific regions within the small and large intestines. For example, epithelial expression of CCL25 directs the colonization of CCR9-expressing IELs to the small intestine, with the most pronounced effects in the proximal duodenum [26–30]. Nonetheless, additional factors that appropriately position specific subsets of IELs remain to be discovered. For instance, upon irradiation-induced damage or infection-induced inflammation, distinct chemokines are produced and the homing requirements of specific IELs change [31, 32]. Moreover, although CCR9-deficiency reduces the seeding of small intestine IELs, these defects are incomplete [26]. Pertussis toxin experiments have suggested that additional Gαi-linked GPRs can partially compensate for CCR9-deficiency, yet the identities of these receptors remain unknown [28].

Through a search for novel chemokine and G-protein coupled receptors that regulate the function of lymphoid progenitors and/or mature lymphocytes, we observed that GPR18 is very highly expressed in IELs. *Gpr18* is well-conserved across species, yet shows limited similarity to its closest paralogs [33, 34]. A screen of ~200 lipid compounds suggested that N-arachidonyl glycine might be an endogenous ligand, as it induced calcium mobilization, chemotaxis, and Gαi signaling in a variety of different GPR18-expressing cell lines [35–39]. Administration of N-arachidonyl glycine led to anti-inflammatory effects in a mouse model of thioglycolate-induced peritonitis, suggesting an immunosuppressive role for GPR18 [40]. Yet other studies found no evidence that N-arachidonyl glycine induces GPR18 activity, at least through canonical pathways [41–43]. As a result of these conflicting studies and the absence of robust genetic tools, the *in vivo* function of GPR18 remains to be fully determined.

GPR18 is primarily expressed in hematopoietic and immune lineages, with particularly high levels in IELs and follicular B cells [44]. To define the role of GPR18 in lymphopoiesis, antibody responses, and IEL development, we generated *Gpr18* ^*-/-*^ mice. Although we observed limited roles in steady-state lymphopoeisis and in antibody responses to virus infections, we found that GPR18 is required for the restoration of small intestine unconventional IELs following bone marrow transplantation. These data are consistent with and expand upon a very recent study using independently generated *Gpr18* ^*-/-*^ mice [43]. Our results demonstrate remarkably specific requirements for GPR18 within distinct IEL subsets.

## Materials and Methods

### Ethics statement

All procedures in this study were specifically approved and carried out in accordance with the guidelines set forth by the Institutional Animal Care and Use Committee at Washington University (approval numbers 20120058, 20120243, and 20140030). Euthanasia was performed by administering carbon dioxide at 1.5L/minute into a 7L chamber until 1 minute after respiration ceased. After this point, cervical dislocation was performed to ensure death.

### Mice

Animals were housed and bred in pathogen-free facilities at Washington University in St. Louis. To generate *Gpr18* deficient mice, *Gpr18* ^*tm1(KOMP)Vlcg*^ targeted C57BL6/N embryonic stem (ES) cells were obtained from the Knockout Mouse Project. Microinjection was carried out at the Washington University Transgenic Knockout Micro-Injection Core. *Gpr18* ^*tm1(KOMP)Vlcg*^ ES cell targeting was confirmed by Southern bot analysis. Prior to lacZ reporter expression analysis, mice were bred to CMV-Cre recombinase mice (The Jackson Laboratory) to eliminate the neomycin resistance cassette. For all other experiments, the neomycin cassette was retained. *Gpr18* ^-/-^ mice were genotyped using primers found on the Knockout Mouse Project website: *Gpr18*-MT, 5’- CGAGTAGTCAGTTAGAGAGG-3’ (forward) and 3’-GTCTGTCCTAGCTTCCTCACTG5’ (reverse); *Gpr18*-WT, 5’-TTACCCAAGCCTCGCTCTG-3’ (forward) and 3’- GCCTTGCCGGTGTTCTTCAG-5’ (reverse). B6.Ly5.2 mice were obtained from NCI and used as CD45.1^+^ recipients, but not as donors in these experiments due to a germline mutation in *Sox13* [45]. Instead, B6.SJL mice from The Jackson Laboratory were used as CD45.1^+^ donors. Wild type C57BL/6J-IgH^a^ mice were obtained from The Jackson Laboratory. Littermate *Gpr18* ^*+/+*^ and *Gpr18* ^*+/-*^ mice were used as controls as indicated. Balb/c, TCRβδ^-/-^, and athymic nude mice were obtained from The Jackson laboratory and have been described previously [46, 47].

### Antibodies and flow cytometry

All processing and staining of cells was carried out in 2% adult bovine serum (Hyclone) in PBS with 1mM EDTA (Sigma). A list of antibodies is provided in S1 Table. Dead cells were excluded by gating out cells that incorporated propidium iodide (Sigma-Aldrich). Cells were sorted and analyzed on a BD FACSAria II or BD LSRII (BD Biosciences). Data was analyzed using FlowJo software (FlowJo, LLC).

### Gpr18 reporter expression analysis

For *Gpr18* reporter analysis, IELs were purified as described from either *Gpr18* ^*+/-*^ *CMV-Cre* ^*+*^ or *Gpr18* ^*+/+*^ mice and analyzed by flow cytometry using the FluoReporter lacZ Flow Cytometry Kit (Molecular Probes) per manufacturer’s instructions.

### Histology

Small intestines were removed from *Gpr18* ^+/+^ or *Gpr18* ^*-/-*^ mice and flushed with 10ml ice cold PBS. Intestines were subsequently flushed with 10ml of 10% neutral-buffered formalin (Sigma-Aldrich), and cut along the anti-mesenteric axis. Intestines were incubated in 10% formalin at 4°C overnight. Intestines were then washed three times with 70% ethanol and then incubated in 70% ethanol overnight at 4°C. After washing with PBS and blocking in 2% agar, intestinal sections were cut and stained with hematoxylin and eosin by the Washington University Developmental Biology Histology and Microscopy Core.

### IEL analysis and isolation

IELs were isolated according to previously published procedures [48]. Small and/or large intestines were removed post-mortem and placed into 5 ml ice cold buffer containing 0.15 M HEPES (Sigma-Aldrich) in Hanks Balanced Salt Solution (Sigma-Aldrich) at pH 7.2. Peyer’s patches were removed, and intestines were opened longitudinally and washed with 3 times with PBS to remove mucus and fecal matter. Intestines were then cut into 2-cm sections, and placed in Hanks Balanced Salt Solution containing 10% fetal bovine serum (Hyclone), 0.015 M HEPES, 0.005 M EDTA at pH 7.2. IELs were isolated by incubating intestinal sections end-over-end for 20 minutes at 37°C two times. The supernatant containing the IELs was centrifuged 5min at 2000g. IELs were further purified via gradient centrifugation by suspending the cells in 44% Percoll (GE Healthcare), overlaying them onto 67% Percoll in PBS and centrifuging for 20 minutes at 2000g at room temperature. The interface containing IELs was isolated, washed and used for further analysis as specified. Gating strategies are provided in S1 Fig.

### Analysis of progenitor cells and mature cells

Femurs, tibiae, humerus, and pelvic bones were isolated and crushed with a mortar and pestle. Leukocytes were isolated by gradient centrifugation for 10min at 2000g using Histopaque 1119 (Sigma-Aldrich). Cells were washed and analyzed by flow cytometry as described above. Gating strategies are provided in S2 and S3 Figs.

### RNA extraction and qRT-PCR analysis

For analysis of *Gpr18* expression in splenocytes, RNA was isolated using TRIzol (Invitrogen) per the manufacturer’s instructions. For analysis of *Gpr18* expression in B cell fractions and progenitors, the specified cells were double-sorted directly into RLT buffer (Qiagen) and RNA was purified using the RNeasy kit (Qiagen) per the manufacturer’s instructions. cDNA was synthesized using Superscript III first-strand synthesis kit (Invitrogen) using random hexamers per the manufacturer’s instructions. Quantitative real-time PCR analysis was carried out using SybrGreen (Applied Biosystems) per the manufacturer’s instructions and an ABI 7000 sequence detection system (Applied Biosystems). The primers used were: *Gpr18*: 5’-GACAGACAGGAGGTTCGACATACA-3’ (forward) 3’-TGTATTCCTCTGGGTGTGGAGCCA-5’ (reverse). Data were normalized to *β-actin*, 5′-gatcattgctcctcctgagc-3′ (forward) and 5′-acatctgctggaaggtggac-3′ (reverse) or *Gapdh*, 5-GGCAAATTCAACGGCACAGT-3′ (forward) and 5-GATGGTGATGGGCTTCCC-3′ (reverse) as specified.

### Generation of bone marrow chimeras

For non-competitive chimeras for influenza infections, 5 x 10^6^ whole bone marrow cells from either *Gpr18* ^+/+^ or *Gpr18* ^*-/-*^ mice were injected into 800 cGy-irradiated B6.Ly5.2 CD45.1^+^ recipients (NCI). For mixed bone marrow chimeras to analyze lineage commitment, 5 x 10^6^ bone marrow cells from either *Gpr18* ^+/+^ or *Gpr18* ^*-/-*^ mice were mixed with 5 x 10^6^ bone marrow cells from B6.SJL CD45.1^+^ (The Jackson Laboratory) mice and injected into 800 cGy-irradiated B6.Ly5.2 CD45.1^+^ recipients. Bone marrow chimeras used to analyze GPR18-dependent antibody responses were generated by transplanting 2 x 10^6^ bone marrow cells from *Gpr18* ^+/+^ or *Gpr18* ^*-/-*^ mice (IgH^b^) with 2 x 10^6^ bone marrow from IgH^a^ mice into 800 cGy-irradiated B6.Ly5.2 CD45.1^+^ recipients. Eight weeks post-transplant, mice were analyzed for chimerism in the specified tissues or used for further experiments. Athymic nude mice were pretreated with a 150μg dose of αCD8α antibody (clone 53–6.7) intraperitoneally 3 days prior to 800 cGy irradiation and transplantation to reduce pre-existing host IELs.

### Allogeneic transplantation and induction of graft versus host disease

All recipient animals in these experiments were 6–7 weeks of age. Allogeneic transplantations were performed as previously described [49, 50]. 5×10^6^ T cell-depleted bone marrow cells (CD45.1^+^ C57Bl/6 (H-2^b^)) and 5×10^5^ splenic pan T cells from CD45.2^+^ *Gpr18* ^*+/+*^ or *Gpr18* ^*-/-*^ (H-2^b^) littermates were intravenously injected into lethally irradiated (900cGy) Balb/c recipient mice (H-2^d^, CD45.2^+^). Sulfamethoxazole and Trimethoprim Oral suspension (Hi-Tech Pharmacal Co., INC., Amityville, NY) (63 ug/ml in drinking water) was provided for the first 10 days post-transplant. Mice were monitored twice per week for the first 4 weeks, and once per week thereafter. Mice were euthanized if 20% of initial body weight was lost. We did not observe any unexpected death in any other mice. 5 mice received *Gpr18* ^*+/+*^ splenocytes, 10 mice received *Gpr18* ^*-/-*^ splenocytes, and 2 mice received only T cell-depleted bone marrow. Analgesics and anesthetics were not administered.

### Immunizations and infections

Mice were immunized with 4-hydroxy-3-nitrophenyl-acetyl conjugated to chicken gamma globulin (NP-CGG) by intraperitoneally injection with 100 μg of NP-CGG (hapten:protein ratio of 15:1 or 16:1; Bioresearch Technologies) that had been precipitated in 5% aluminum potassium hydroxide (Sigma) in PBS. Mice were bled at 2, 4, and 8 weeks post-immunization and serum was analyzed for NP-specific antibodies by ELISA. West Nile virus strain New York 1999 infections and serological analyses were performed as previously described [51]. Mice were infected in the footpad with 10^2^ plaque-forming units (PFU) of virus, and serum was collected 8 weeks post-infection. For influenza infections, 10 *Gpr18* ^+/+^ or 10 *Gpr18* ^*-/-*^ bone marrow chimeric mice of 16 weeks of age were infected intranasally with the influenza virus A/Puerto Rico/8/34 (A/PR/8/34) at a dose of 500 egg infective dose 50. Weight was monitored at regular intervals before and for 20 days after virus infection. No mice died during the course of these experiments and all animals lost <20% of their initial weight. For *C*. *rodentium* infections, 10 *Gpr18* ^*+/+*^ and 10 *Gpr18* ^*-/-*^ bone marrow chimeras of 16 weeks of age received 2 x 10^9^ strain DBS100 (American Type Culture Collection) bacteria via oral gavage. Mice were weighed every 2–3 days for 37 days post-infection. Mice were euthanized if 20% of the initial body weight was lost. Two *Gpr18* ^*-/-*^ bone marrow chimeras were euthanized at days 16 and 21 in accordance with this criterion. No other mice died unexpectedly in these experiments. Analgesics and anesthetics were not administered.

### Serological analysis

ELISA plates were coated with 5 μg/ml NP_16_ or NP_4_ or with WNV envelope (E) protein overnight at 4°C in coating buffer containing 0.1 M sodium bicarbonate and 0.02% sodium azide at pH 9.6. Plates were washed three times with ELISA wash buffer (0.05% Tween 20 in PBS). Plates were blocked for 1 hour at room temperature with 2% BSA/PBS blocking buffer. Sera was then serially diluted in blocking buffer, plated and incubated for 1 hour at room temperature. Plates were washed three times followed by incubation with 1μg/ml biotinylated anti-mouse IgG (Jackson Immunoresearch), anti-IgG1^a-^, or anti-IgG1^b^ (BD Biosciences) for 1 hour at room temperature. Wells were washed three times and then streptavidin-conjugated horseradish peroxidase (BD Biosciences) was added to each well and incubated for 1 hour at room temperature. Wells were washed three times with wash buffer and one time with PBS. Peroxidase activity was detected using tetramethylbenzidine (Dako) and quenched with 2 N H_2_SO_4_. Optical densities were measured at 450 nm. The end-point titer of each sample was calculated by a one-phase exponential decay curve and defined as the dilution that generated an OD_450_ value that was 3 standard deviations above background. Prism software (GraphPad Software) was used to calculate the end-point titer.

## Results

**Fig 1:**
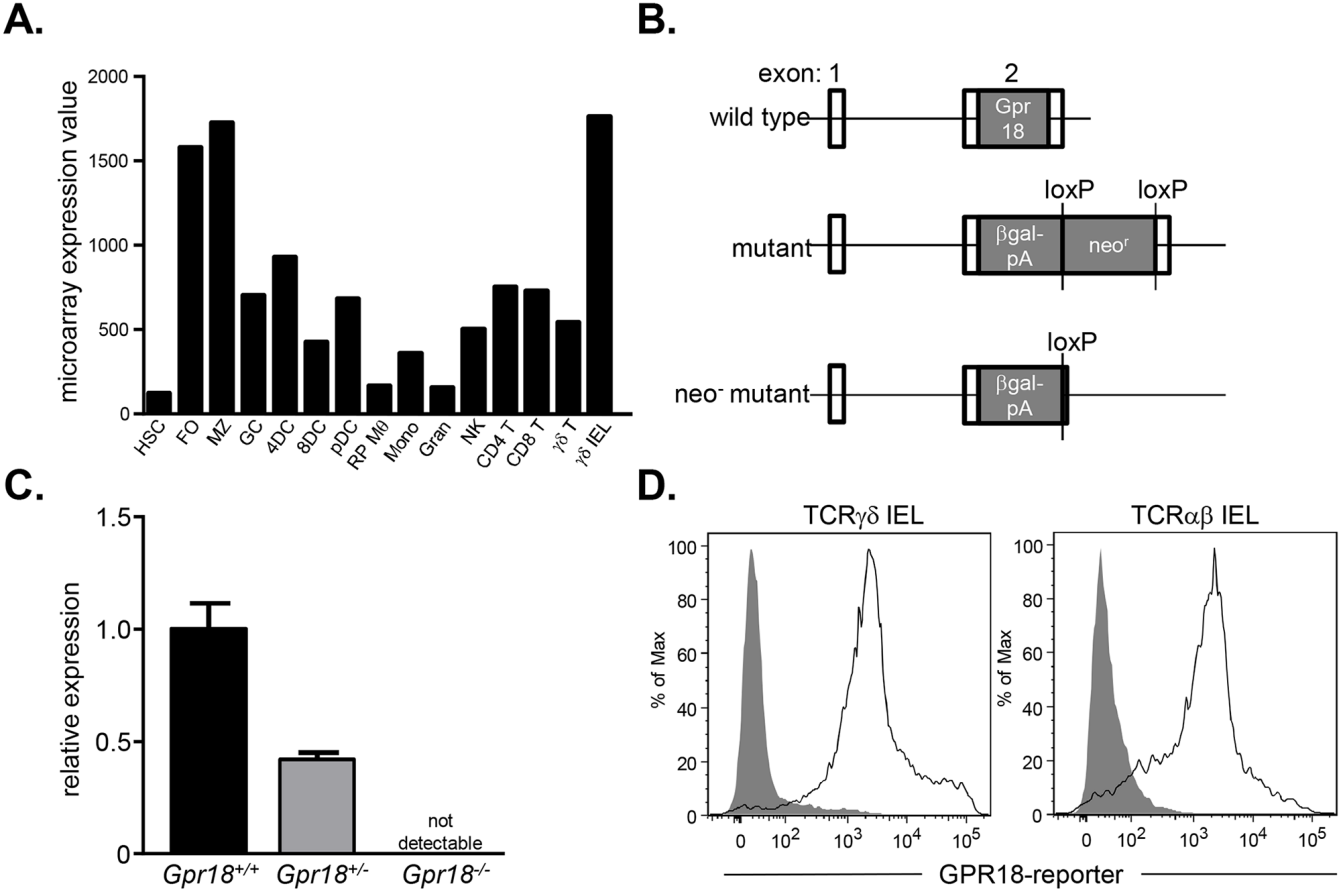
GPR18 is highly expressed by intestinal IELs. (A) Microarray expression data obtained from the Immunological Genome Project. (B) Targeting strategy for *Gpr18*-deficient alleles. For reporter assays, the neomycin cassette was deleted by crossing *Gpr18* ^*+/-*^ mice to a CMV-Cre-expressing strain. (C) Quantitative RT-PCR analysis of GPR18 expression in *Gpr18* ^*+/+*^ (black bar), *Gpr18* ^*+/-*^ (gray bar), and *Gpr18* ^*-/-*^ splenocytes (not detectable, so not visible on graph). Reactions were performed in technical triplicates and data are normalized to β-actin expression. Mean values ± SEM are shown. (D) Flow cytometry analysis of GPR18 expression through β-galactosidase expression in small intestine TCRγδ^+^ and TCRαβ^+^ IELs. Gating strategies are shown in S1 Fig. Cells from heterozygous *Gpr18* ^*+/-*^ CMV-Cre^+^ (solid lines) and control *Gpr18* ^*+/+*^ (filled histogram) mice were stained with the β-galactosidase substrate fluorescein di-β-galactopyranoside. HSC, hematopoietic stem cell; FO, follicular B cell; MZ, marginal zone B cell; GC, germinal center B cell; 4DC, CD4^+^ splenic dendritic cell; 8DC, CD8^+^ splenic dendritic cell; pDC, plasmacytoid dendritic cell; RP Mθ, red pulp macrophage; Mono, bone marrow Ly6c^-^ monocyte; Gran, bone marrow granulocyte; NK, splenic natural killer cell; CD4 T, CD4^+^ T cell; CD8 T, CD8^+^ T cell, γδ T, splenic TCRγδ T cell; γδ IEL, TCRγδ^+^ intestinal IEL.

Intestinal IELs are localized to gain access to luminal antigens and maintain contact with enterocytes. The full complement of homing receptors used by IELs for proper positioning is unknown. We thus examined datasets from the Immunological Genome Project for chemokine and G-protein coupled receptors preferentially expressed by IELs. These analyses revealed that TCRγδ^+^ intestinal IELs, but not splenic TCRγδ^+^ cells, express high levels of *Gpr18* (Fig 1A). To test the physiological importance of elevated *Gpr18* expression in IELs, we generated *Gpr18* ^*-/-*^ mice. C57BL6/N mouse embryonic stem (ES) cells carrying a deletion of the entire coding region of *Gpr18* were obtained from the Knockout Mouse Project. In these ES cells, *Gpr18* is replaced with a β-galactosidase cassette and a floxed neomycin resistance gene (Fig 1B). Using these ES cells, *Gpr18* ^*+/-*^ mice were generated. Heterozygous mice were intercrossed to generate *Gpr18* ^*-/-*^ mice and control littermates. Quantitative RT-PCR analysis of splenic RNA confirmed the complete loss of *Gpr18* transcripts in *Gpr18* ^*-/-*^ animals, and a 50% reduction in transcript levels in *Gpr18* ^*+/-*^ animals relative to wild type littermates (Fig 1C). To assess *Gpr18* expression in IELs, we crossed *Gpr18* ^*+/-*^ mice to Cre recombinase-expressing animals to remove the neomycin resistance cassette (Fig 1B). The β-galactosidase activity was then used as a transcriptional reporter for GPR18 and measured through cleavage of the substrate fluorescein di-β-galactopyranoside [52]. Analysis of GPR18 using this reporter confirmed expression in both TCRγδ^+^ and TCRαβ^+^ small intestine IELs (Fig 1D). Thus, GPR18 is highly expressed in intestinal IELs, and the functional importance of this gene can be tested with the *Gpr18* ^*-/-*^ animals.

**Fig 2:**
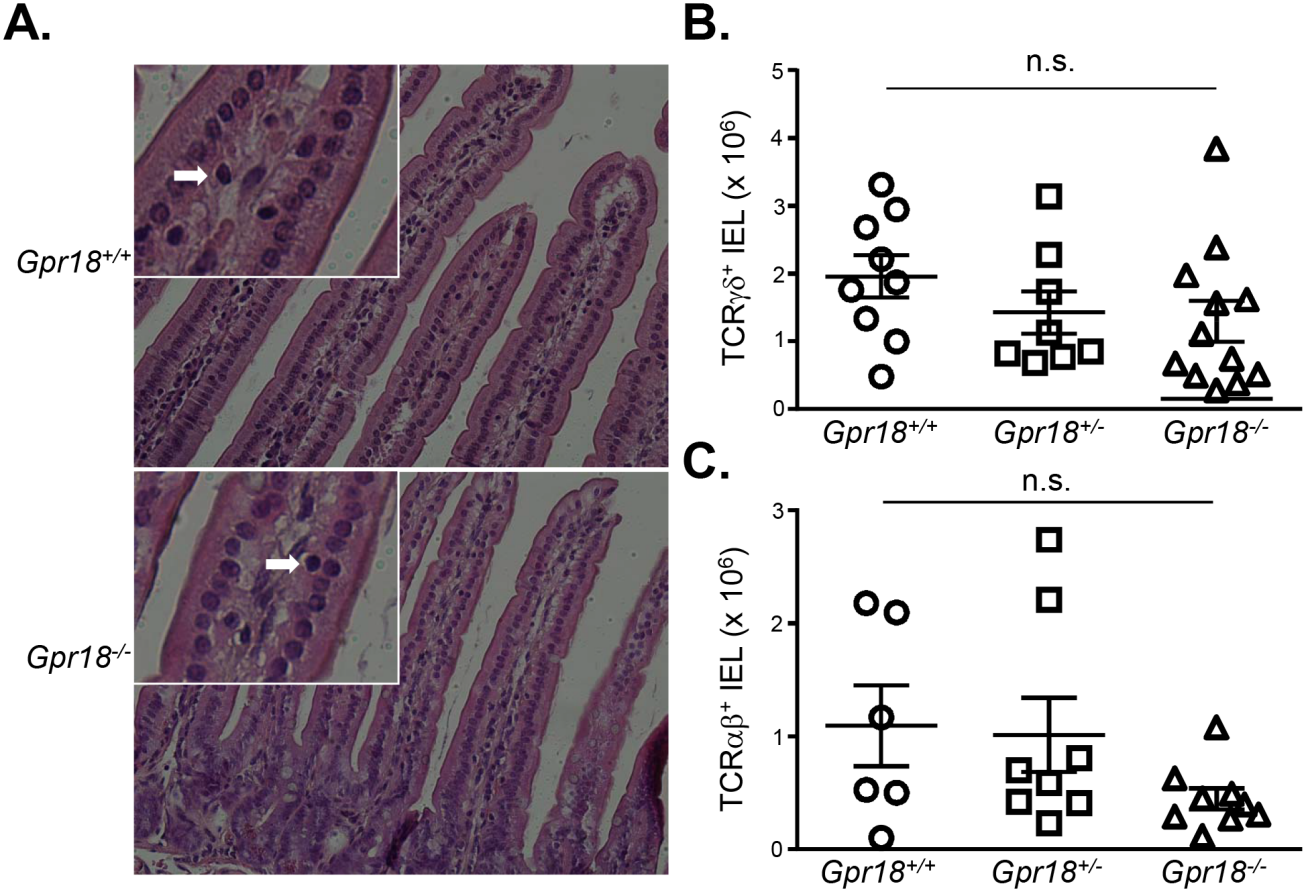
GPR18 is not required for steady state intestinal IELs. (A) H&E sections of small intestine from *Gpr18* ^*+/+*^ and *Gpr18* ^*-/-*^ mice. White arrows depict examples of IELs. Insets show relevant portions at higher magnification. Sections were obtained from duodenum of small intestine, and are representative of 2 mice. (B) Numerical analysis of small intestine IELs from *Gpr18* ^*+/+*^, *Gpr18* ^*+/-*^, and *Gpr18* ^*-/-*^ mice. Frequencies of TCRγδ (B) and TCRαβ (C) IELs were determined by flow cytometry, and IEL absolute numbers were calculated using the total recovered cell number from the small intestine. Mean values ± SEM are shown. Each symbol represents one mouse. n.s., not significant using 1-way ANOVA.

To begin to assess the impact of GPR18 deficiency on IELs, we histologically examined intestines. Hematoxylin and eosin-stained sections revealed grossly normal intestinal structure and organization in *Gpr18* ^*-/-*^ mice. Villous size and morphology appeared similar between *Gpr18* ^*-/-*^ mice and wild type littermates, and small intestine IELs were readily apparent in both groups (Fig 2A). To quantify IEL numbers, we performed flow cytometric analysis. These results revealed similar numbers of TCRγδ^+^ IELs in *Gpr18* ^*+/+*^, *Gpr18* ^*+/-*^, and *Gpr18* ^*-/-*^ mice (Fig 2B). Analysis of *Gpr18* ^*+/+*^, *Gpr18* ^*+/-*^, and *Gpr18* ^*-/-*^ TCRαβ^+^ IELs also revealed no defects (Fig 2C).

**Fig 3:**
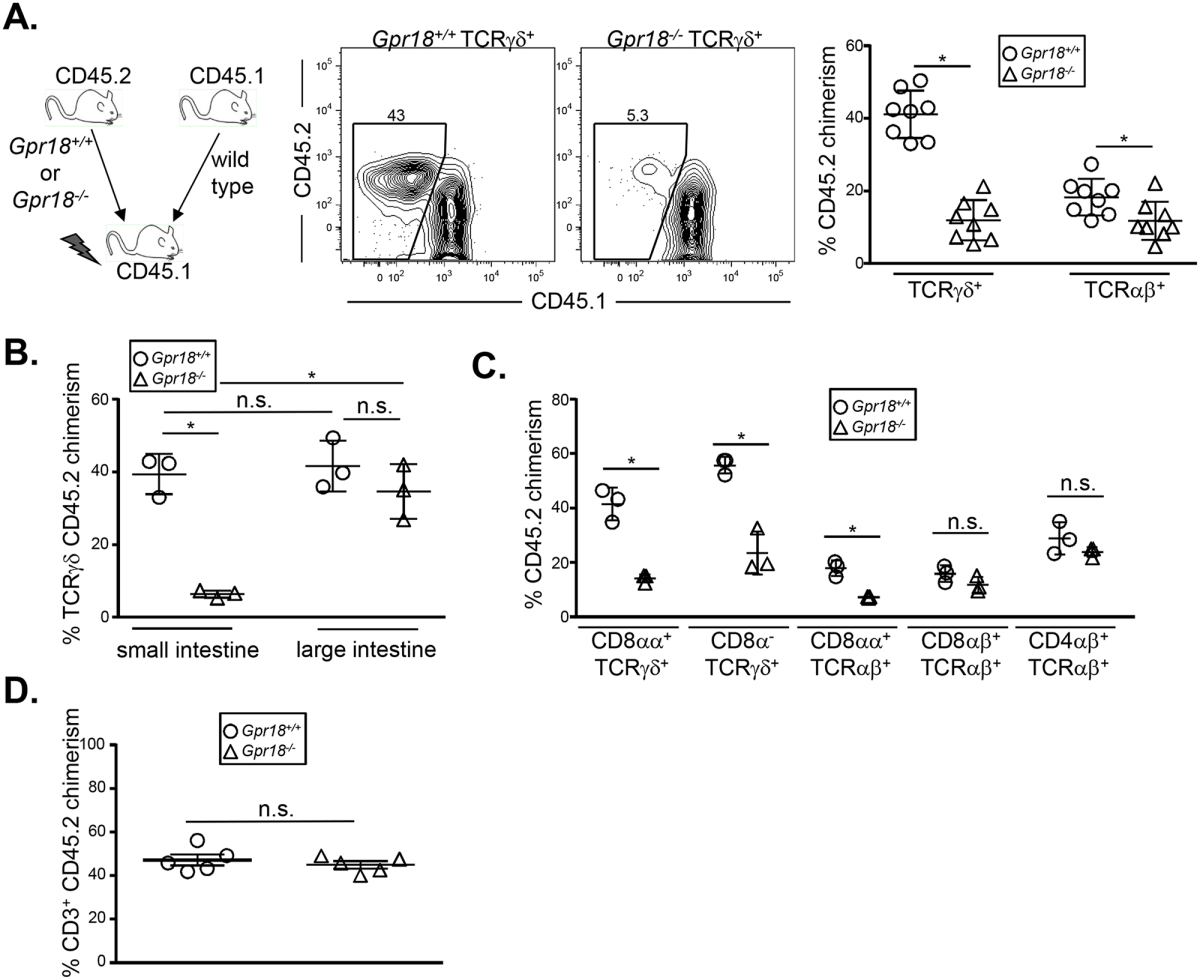
GPR18 is essential for optimal IEL reconstitution following bone marrow transplantation. Donor chimerism analysis of T cells in the intestine and spleen. Equal numbers of wild type CD45.1^+^ and either *Gpr18* ^*+/+*^ or *Gpr18* ^*-/-*^ CD45.2^+^ bone marrow cells were transplanted into 800cGy-irradiated CD45.1^+^ recipients. Eight weeks after reconstitution, small (A, C) or both small and large (B) intestines were harvested and donor CD45.2^+^ chimerism in TCRγδ and TCRαβ (A, B) and specific IEL subsets (C) were analyzed by flow cytometry. (D) Total splenic T cell chimerism is shown. Gating strategies are shown in S1 Fig. Mean values ± SEM are shown. *p<0.05 using student's 2-tailed unpaired t-test.

Incomplete defects in immune lineage development can often be masked and corrected through homeostatic expansion of the affected cell type. Such defects can often be revealed through competitive transplantation experiments. For example, CCR9-deficient mice show modest IEL defects under steady-state conditions, but *Ccr9* ^*-/-*^ bone marrow reconstitutes small intestine IELs poorly in competitive transplantation experiments [26]. To determine if cell-intrinsic defects in *Gpr18* ^*-/-*^ IELs could be observed under competitive settings, we mixed equal numbers of wild type CD45.1^+^ bone marrow cells with CD45.2^+^ *Gpr18* ^*+/+*^ or *Gpr18* ^*-/-*^ bone marrow. This mixture was then transplanted into 800 cGy-irradiated CD45.1^+^ recipients. At 8 weeks post-transplantation, we analyzed chimerism of TCRγδ^+^ and TCRαβ^+^ small intestine IELs. Strikingly, *Gpr18* ^*-/-*^ cells contributed poorly to the TCRγδ^+^ IEL compartment (Fig 3A). A statistically significant defect was also observed in the TCRαβ^+^ compartment, although the overall donor contribution was low for both *Gpr18* ^*+/+*^ and *Gpr18* ^*-/-*^ groups, perhaps due to the persistence of radioresistant CD45.1^+^ host cells (Fig 3A). In preliminary studies, we have also found that *Gpr18* ^*-/-*^ bone marrow poorly reconstitutes small intestine IELs when competed against wild type bone marrow in unirradiated TCRβδ^-/-^ recipients, suggesting that irradiation-induced damage is not necessary to elicit the observed phenotype. Thus, GPR18 is required for optimal restoration of small intestine IELs following transplantation.

Small and large intestine IELs have different homing requirements. For example, CCR9 is important for small intestine IEL recruitment, but is dispensable for large intestine IELs [26]. To determine if GPR18 is selectively required for reconstitution of small intestine IELs, we generated mixed chimeras and analyzed IEL chimerism in both the small and large intestines. We again observed poor reconstitution of small intestine TCRγδ^+^ IEL by *Gpr18* ^*-/-*^ bone marrow, but large intestine TCRγδ^+^ IELs were restored normally (Fig 3B). Thus, GPR18 is selectively required for the optimal reconstitution of small intestine IELs.

Intestinal IELs can be subdivided on TCR γδ vs αβ usage, and also upon expression of the coreceptors CD8αα, CD8αβ, and CD4 [53]. To determine if GPR18 is selectively required by one or more of these subsets following bone marrow transplantation, we analyzed chimerism in the small intestine. CD8αα^+^ TCRγδ small intestine IELs were reconstituted poorly by GPR18-deficient bone marrow (Fig 3C). CD8αα^+^ TCRαβ^+^ IELs were also regenerated at lower levels by *Gpr18* ^*-/-*^ bone marrow, whereas conventional CD4 TCRαβ^+^ IELs were reconstituted normally (Fig 3C). *Gpr18* ^*-/-*^ CD8α^-^ TCRγδ and CD8αβ^+^ TCRαβ^+^ IELs also appeared reduced (Fig 3C), although this was not consistent across all independent experiments. T cell contribution in the spleen was identical between *Gpr18* ^*+/+*^ and *Gpr18* ^*-/-*^ donors, excluding a general role for GPR18 in T cell reconstitution (Fig 3D). Thus, GPR18 is specifically required for the restoration of CD8αα^+^ TCRγδ and TCRαβ^+^ IELs in the small intestine.

**Fig 4:**
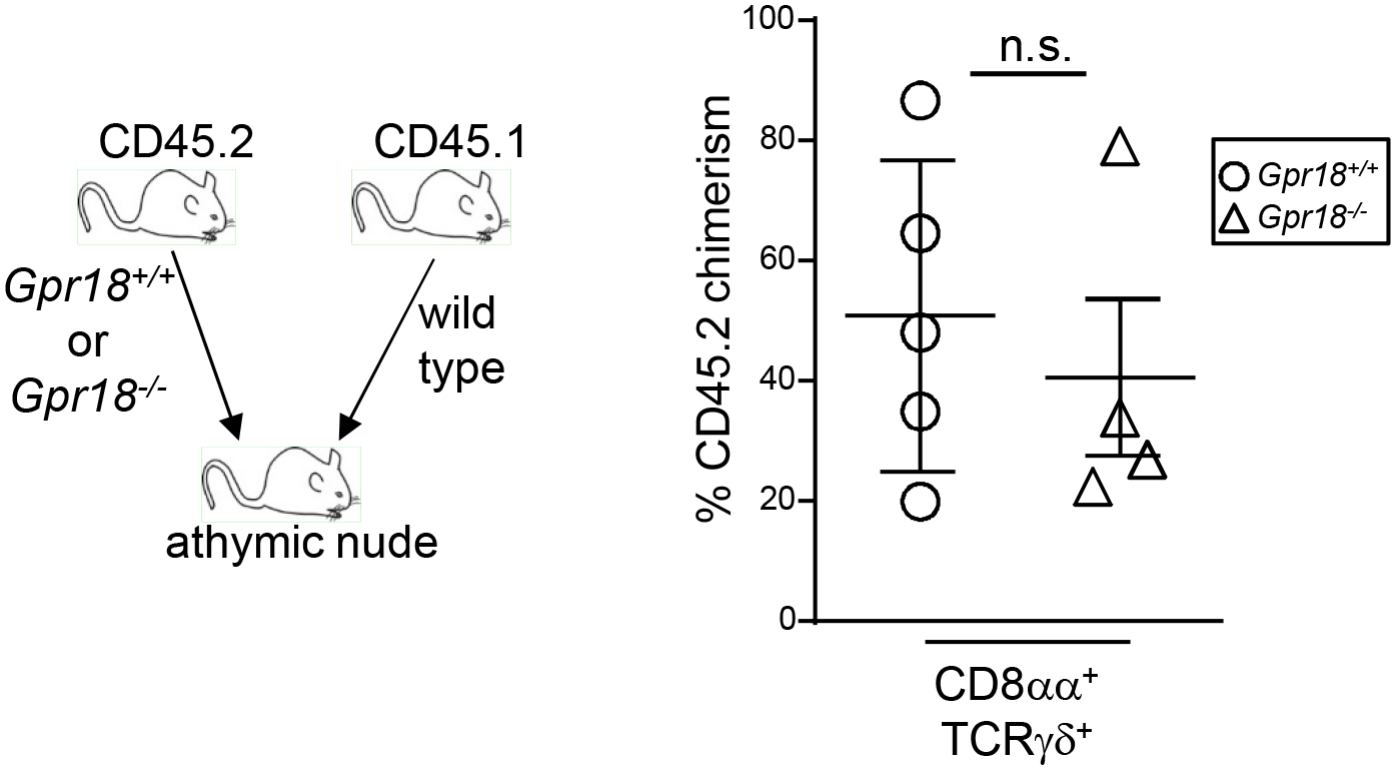
GPR18 is essential for competitive reconstitution of thymus-derived IELs irrespective or irradiation. Equal numbers of wild type CD45.1^+^ and either *Gpr18* ^*+/+*^ or *Gpr18* ^*-/-*^ CD45.2^+^ bone marrow cells were transplanted into irradiated athymic nude mice. Eight weeks post-transplantation, CD45.2^+^ donor chimerism of CD8αα^+^ TCRγδ^+^ IELs were analyzed in the small intestine by flow cytometry. Mean values ± SEM are shown. *p<0.05 using student's 2-tailed unpaired t-test.

The dominant pathway of IEL development is through the thymus [23, 24, 31, 54]. However, CD8αα^+^ TCRγδ^+^ IELs can be found in athymic nude mice [55, 56]. To determine if GPR18 is also required for the athymic pathway of CD8αα^+^ TCRγδ^+^ IEL development, we performed competitive bone marrow reconstitutions of irradiated athymic nude mice. Under these settings, *Gpr18* ^*-/-*^ bone marrow reconstituted small intestine IELs to similar levels as did *Gpr18* ^*+/+*^ bone marrow (Fig 4). These data suggest that GPR18 is specifically required for thymic reconstitution of IELs.

**Fig 5:**
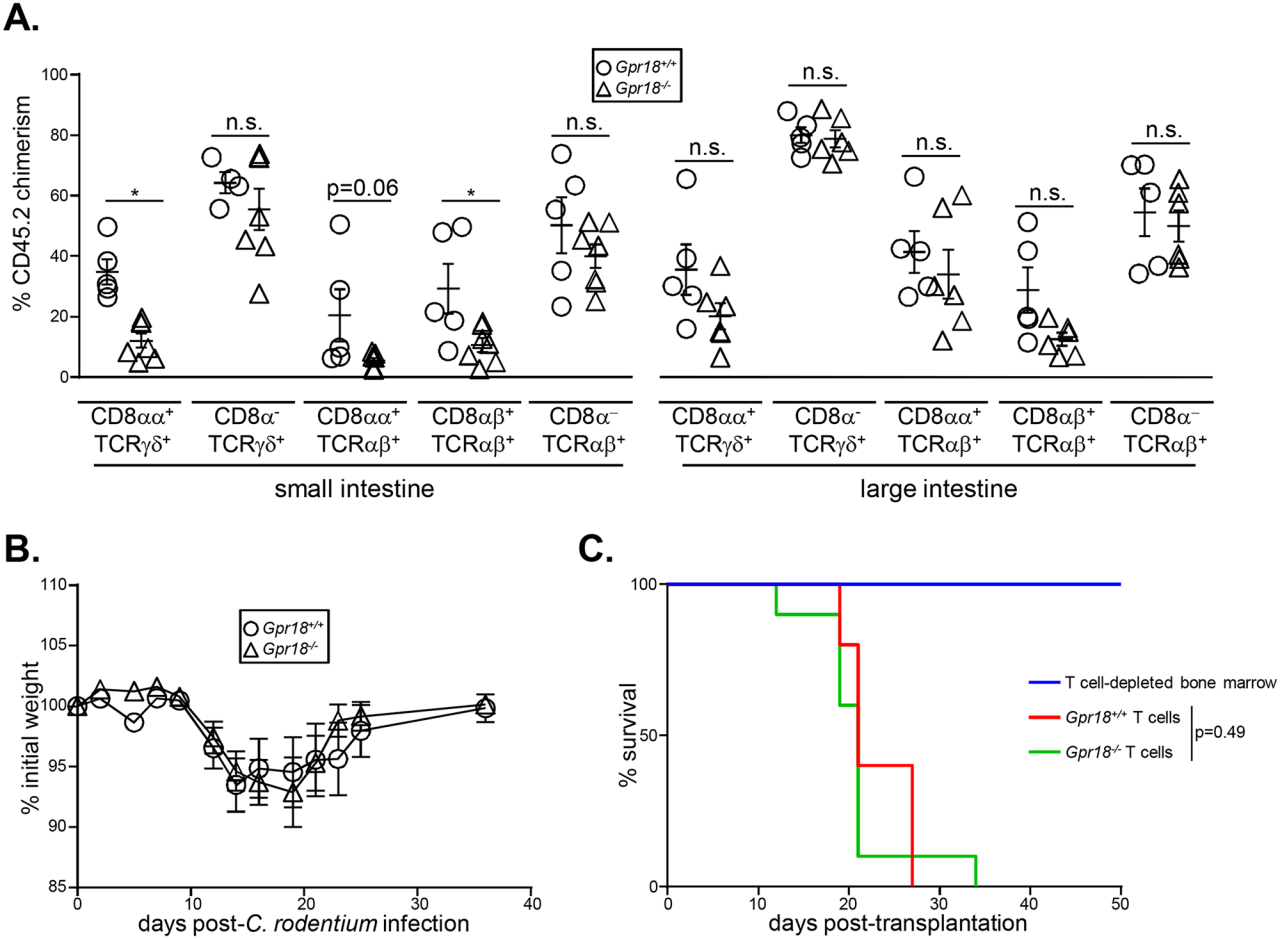
GPR18 is dispensable for resistance to *C*. *rodentium* infections and graft versus host disease. (A) 5 x 10^6^ bone marrow cells from *Gpr18* ^*-/-*^ or *Gpr18* ^*+/+*^ mice were transplanted into sublethally irradiated wild type CD45.1 recipients. Intestinal IEL chimerism was measured 8 weeks post-transplantation. Mean values ± SEM are shown. *p<0.05 using student's 2-tailed unpaired t-test. (B) *Gpr18* ^*-/-*^ or *Gpr18* ^*+/+*^ bone marrow chimeras were orally gavaged with *C*. *rodentium*. Weight after infection was quantified for 37 days. Mean values ± SEM are shown, n = 10 for each group. No statistically significant differences were observed at any timepoint by students' 2-tailed t test. (C) Lethally-irradiated (900 cGy) Balb/c mice were transplanted with 5 x 10^6^ T cell-depleted bone marrow cells from a CD45.1^+^ C57Bl/6 mice with or without 5 x 10^5^ splenic pan T cells from CD45.2^+^ *Gpr18* ^*+/+*^ or *Gpr18* ^*-/-*^ littermates. Survival was measured over the course of 50 days. Two recipients of T cell-depleted bone marrow only, 10 recipients of *Gpr18* ^*-/-*^ splenic pan T cells, and 5 recipients of *Gpr18* ^*+/+*^ splenic pan T cells were assessed. No significant differences (p = 0.49) in survival were observed using log-rank Mantel-Cox test.

Given the role of GPR18 in reconstituting IELs following transplantation, we reasoned that *Gpr18* ^*-/-*^ bone marrow chimeras may have functional defects in response to intestinal insults. First, we determined that *Gpr18* ^*-/-*^ bone marrow poorly reconstitutes small intestine CD8αα^+^ TCRγδ^+^ and CD8αβ^+^ TCRαβ^+^ IELs even in the absence of co-transplanted wild type bone marrow, perhaps due to competition with residual radioresistant IELs (Fig 5A). In contrast, all subsets of large intestine IELs were reconstituted normally (Fig 5A). We next challenged *Gpr18* ^*-/-*^ or control chimeras with *Citrobacter rodentium*, a model of enteropathogenic *E*. *coli* infections [57]. *C*. *rodentium* infections primarily affect the large intestinal epithelia, but damage to the small intestine also occurs and correlates with diarrhea and weight loss [58]. Weight loss and gain were thus measured over the course of 37 days post-infection. No differences at any timepoint were observed between mice reconstituted with *Gpr18* ^*+/+*^ or *Gpr18* ^*-/-*^ bone marrow (Fig 5B). These data indicate that despite a diminished IEL compartment, *Gpr18* ^*-/-*^ chimeras respond normally to *C*. *rodentium* infection.

We next sought to test the importance of GPR18 in the context of graft versus host disease (GvHD). During GvHD, alloreactive donor T cells attack host tissues such as skin, liver, and intestinal epithelia following allogeneic bone marrow transplantation. Epithelial damage and microbial dysbiosis contribute to the severity of GvHD, as germ-free mice develop only attenuated disease [59]. Both host- and donor-derived IELs play an important pathogenic role in GvHD, driven by the accumulation of FasL-expressing cells which mediate the cytolysis of epithelial cells [60–64]. To assess the role of GPR18 in GvHD, we used an MHC-mismatched bone marrow transplantation model in which intestinal GvHD is the primary cause of lethality. Lethally-irradiated (900 cGy) H-2^d^ Balb/c mice were transplanted with T cell depleted bone marrow cells (5 x 10^6^ cells) from wild type H-2^b^ CD45.1^+^ C57Bl/6 mice and 5 x 10^5^ splenic pan T cells from H-2^b^ CD45.2^+^ C57Bl/6 *Gpr18* ^*+/+*^ or *Gpr18* ^*-/-*^ mice. Mortality was measured over the course of 50 days. No significant differences in survival were observed between recipients of wild type or *Gpr18* ^*-/-*^ splenic T cells (Fig 5C). Thus, GPR18 is dispensable for cytotoxicity of alloreactive T cells in the context of GvHD. However, it is possible that GPR18 is functionally required in other contexts.

**Fig 6:**
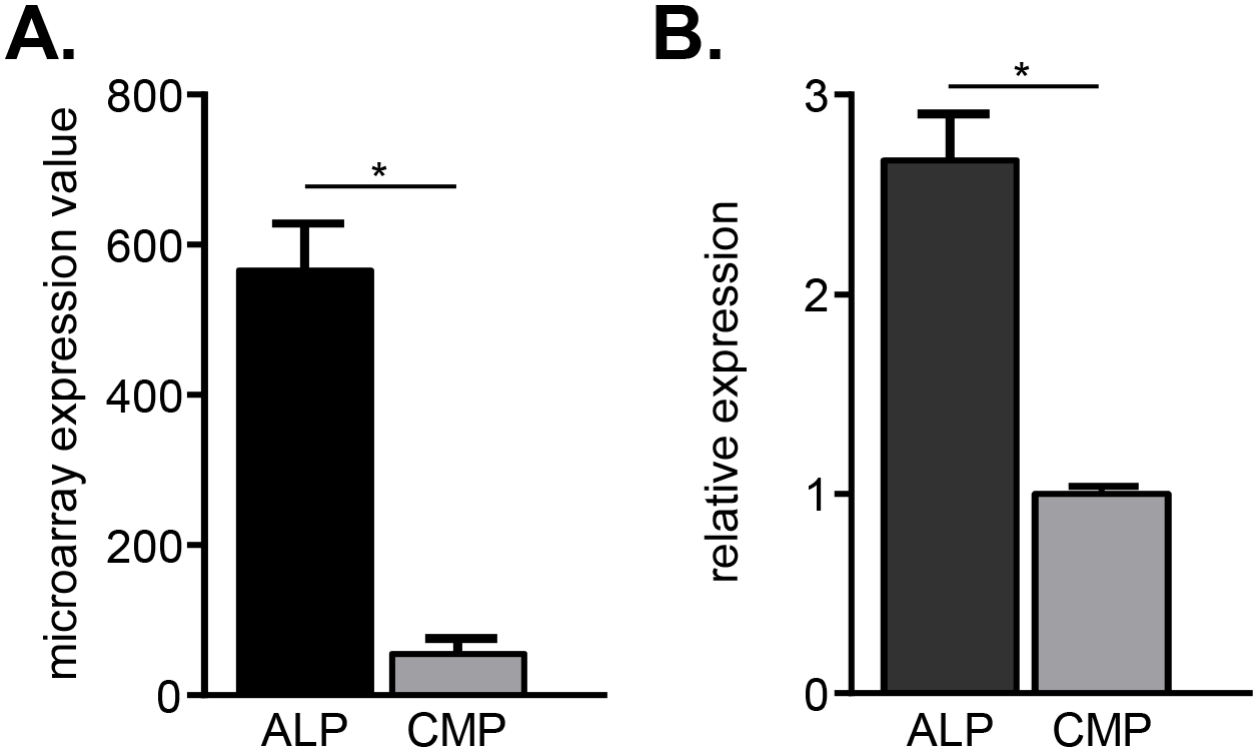
GPR18 is preferentially expressed by lymphoid vs. myeloid progenitors. (A) Microarray expression analysis of GPR18 in all lymphoid progenitors (ALP) and Flk2^+^ common myeloid progenitors (CMP). Mean values ± SEM are shown; n = 3 microarrays each, derived from previously published work [65, 66]. (B) Quantitative RT-PCR analysis of GPR18 in ALP and Flk2^+^ common myeloid progenitors CMP. Mean values ± SEM are shown. Gating strategies are shown in S2 Fig. Data are normalized to *Gapdh* expression levels. *p<0.05 using students 2-tailed unpaired t-test.

We therefore sought to test the specificity of GPR18 function in additional cell types. Analysis of our previous microarray data of hematopoietic progenitors revealed interesting expression patterns of GPR18, suggesting a possible role in lympho- and myelopoiesis [65, 66]. Using these microarray data, we observed that all-lymphoid progenitors (ALPs) express GPR18 at 10-fold higher levels than do Flk2+ common myeloid progenitors (CMPs) (Fig 6A). To confirm this expression difference, we performed qRT-PCR analysis for GPR18 transcripts in ALPs and CMPs. These data demonstrated a nearly 3-fold increase in GPR18 expression in ALPs relative to CMPs (Fig 6B). Thus, GPR18 is more highly expressed in ALPs than in CMPs, and we hypothesized that it may be involved in controlling progenitor cell behavior *in vivo*.

**Fig 7:**
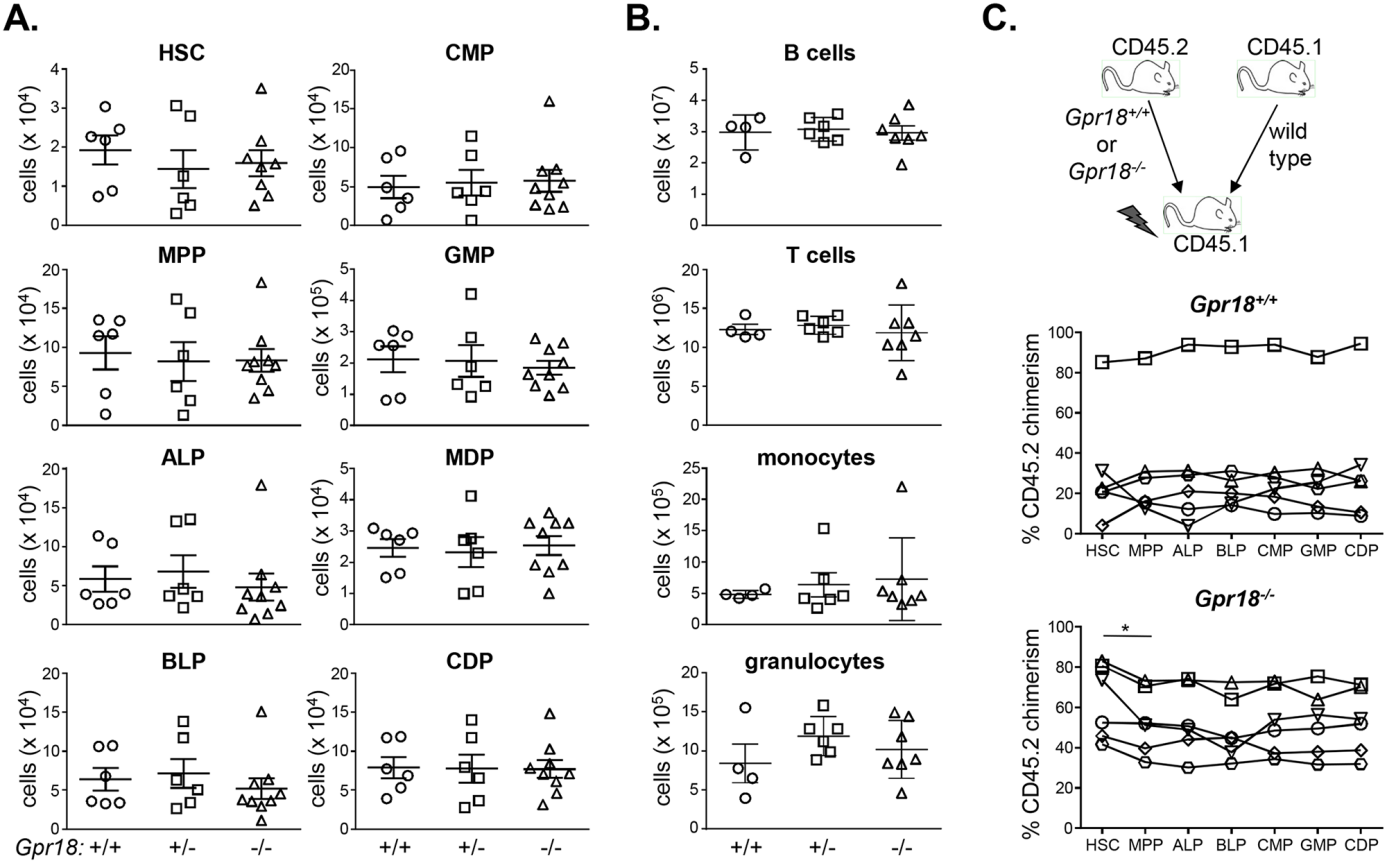
GPR18 is not required for lymphopoiesis or myelopoiesis. (A) Hematopoietic stem cell and progenitor numbers were quantified in *Gpr18* ^*+/+*^, *Gpr18* ^*+/-*^, and *Gpr18* ^*-/-*^ littermates. Mean values ± SEM are shown. No significant differences were observed between any genotype in any progenitor analyzed using 1-way ANOVA. Data are cumulative of 3 independent experiments. Gating strategies are shown in S2 Fig. (B) Mature splenic B and T cells, monocytes, and granulocytes were quantified in *Gpr18* ^*+/+*^, *Gpr18* ^*+/-*^, and *Gpr18* ^*-/-*^ mice. No significant differences were observed between any genotype in any progenitor analyzed using 1-way ANOVA. Gating strategies are shown in S3 Fig. (C) Donor chimerism analysis of bone marrow progenitors. Equal numbers of wild type CD45.1^+^ and *Gpr18* ^*+/+*^ or *Gpr18* ^*-/-*^ CD45.2^+^ bone marrow cells were transplanted into 800cGy-irradiated CD45.1^+^ recipients. Eight weeks after reconstitution, CD45.2^+^ chimerism was quantified in progenitors as gated in (A) followed by analysis of CD45.1 and CD45.2 expression. Each unique symbol connected by a line represents chimerism within an individual mouse. *p<0.05 using student's 2-tailed paired t-test. No other significant differences were observed in downstream progenitors relative to upstream MPPs. HSC, hematopoietic stem cell; MPP, multipotent progenitor; ALP, all-lymphoid progenitor; BLP, B lymphoid progenitor; CMP, common myeloid progenitor; GMP, granulocyte macrophage progenitor; MDP, monocyte dendritic cell progenitor; CDP, common dendritic cell progenitor.

Lymphoid progenitors generate few myeloid cells *in vivo*, yet readily do so *in vitro* when exposed to physiological concentrations of myeloid-promoting cytokines, such as M-CSF [67–71]. These lymphoid progenitors may prevent their output *in vivo* by homing to specific niches which limit their exposure to myeloid-promoting factors [72–74]. Consistent with this possibility, lymphoid-biased progenitors generate more myeloid cells *in vivo* when exposed to Pertussis toxin, an inhibitor of Gαi-coupled receptors and chemotaxis [75]. Given its elevated expression in ALPs relative to CMPs, we reasoned that GPR18 may represent such a Gαi-coupled receptor. To determine the physiological importance of elevated GPR18 expression in ALPs and hematopoiesis, we first analyzed hematopoietic progenitor cells in *Gpr18* ^*-/-*^ mice and littermate controls. The numbers of *Gpr18* ^*-/-*^ hematopoietic stem cells (HSCs), multipotent progenitors (MPPs), ALPs, and B lymphoid progenitors (BLPs) were unchanged relative to *Gpr18* ^*+/+*^ controls (Fig 7A). Similarly, myeloid progenitors including CMPs, granulocyte-macrophage progenitors (GMPs), macrophage-dendritic cell progenitors (MDPs), and common dendritic cell progenitors also were unaffected by a deficiency of GPR18 (Fig 7A). Thus, gross changes were not observed in hematopoietic development in *Gpr18* ^*-/-*^ mice.

Although the numbers of progenitors in *Gpr18* ^*-/-*^ mice were unaltered, we reasoned that the functional output of these progenitors might be compromised. In this case, changes in the mature cell numbers in peripheral organs might be observed. To address this possibility, we quantified the numbers of B cells, T cells, monocytes, and granulocytes in the spleen (Fig 7B) and bone marrow. Again, no deficits were seen in the numbers of these mature cells in *Gpr18* ^*-/-*^ mice (Fig 7B). Thus, GPR18 is not required for steady-state lympho- or myelopoeisis.

As with the IELs, we postulated that incomplete progenitor defects might be observed in competitive transplantation settings. For example, the transcription factor IRF8 regulates commitment to the dendritic cell lineage, but defects in the formation of the earliest *Irf8* ^*-/-*^committed dendritic cell precursor are apparent only in competitive transplantation settings [66]. To determine if similar defects would be observed for GPR18-deficiency, we performed competitive bone marrow transplantation experiments. Wild type CD45.1^+^ bone marrow was mixed with equal numbers of CD45.2^+^ *Gpr18* ^*+/+*^ or *Gpr18* ^*-/-*^ bone marrow and transplanted into 800 cGy-irradiated CD45.1^+^ recipients. CD45.2^+^ chimerism across progenitor stages was then quantified relative to the upstream HSC chimerism in each animal. This analysis normalizes to the initial engraftment, and is a sensitive measure of any downstream stage-specific defects. As expected, donor *Gpr18* ^*+/+*^ cells contributed proportionately to the HSC chimerism at each stage of development (Fig 7C). *Gpr18* ^*-/-*^ cells showed a small, but statistically significant reduction in chimerism from HSCs to MPPs, a stage through which all hematopoietic cells traverse (Fig 7C) [76, 77]. As GPR18 is expressed at only low levels in both HSCs and MPPs [78], the basis and physiological importance of this decrease is unclear. No subsequent developmental changes in any progenitors were observed relative to the *Gpr18* ^*-/-*^ MPP chimerism (Fig 7C). Thus, GPR18-deficiency minimally affects hematopoietic development. Nonetheless, these data do not exclude a role for GPR18 in downstream functional immune responses.

**Fig 8:**
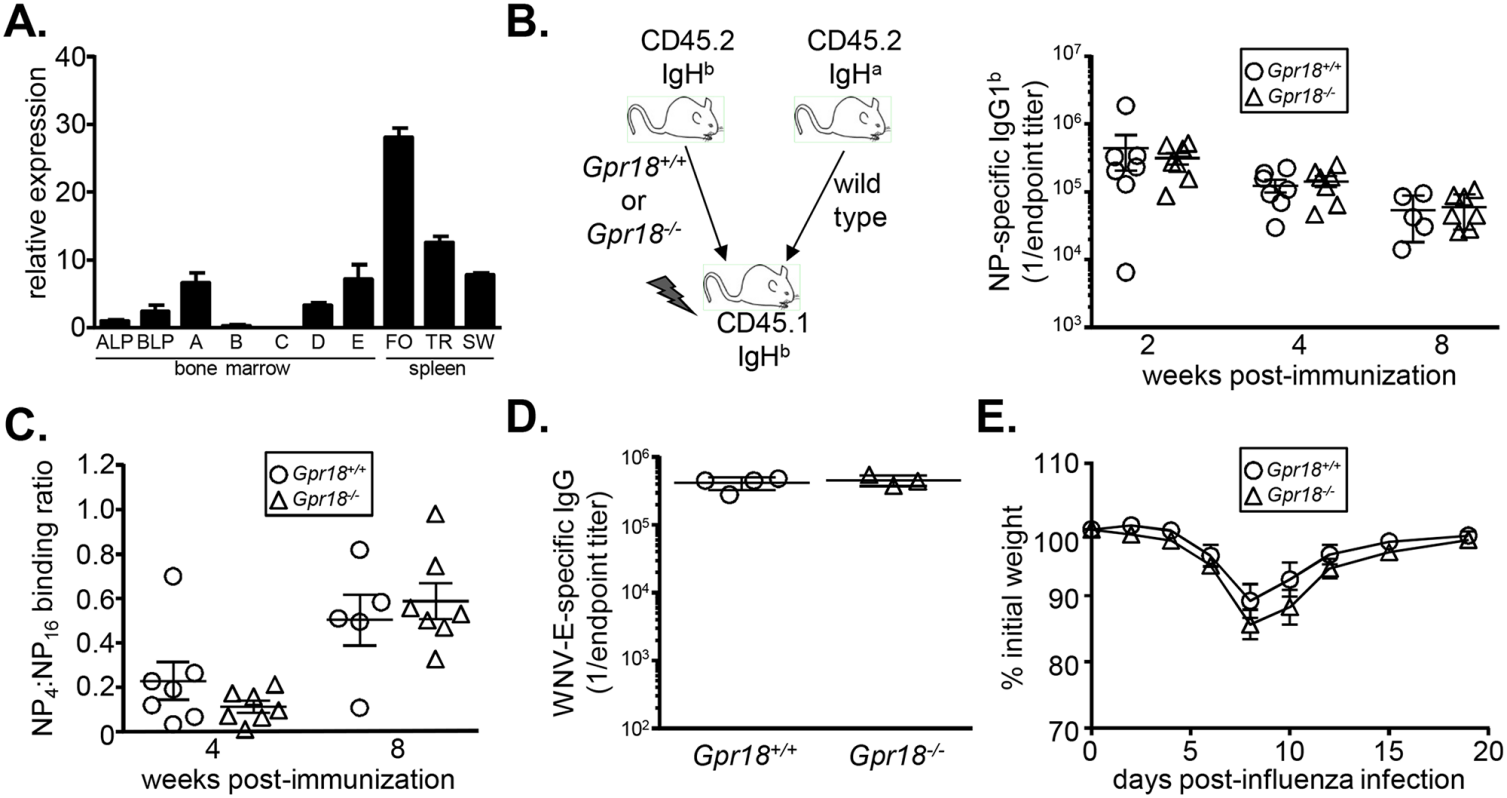
GPR18 is not required for antibody responses. (A) Quantitative RT-PCR analysis of GPR18 expression in B cell progenitors and mature subsets. Data are normalized to *Gapdh* expression. Mean values ± SEM are shown; n = 3. Data are representative of 2 independent experiments. Gating strategies are shown in S2 and S3 Figs. (B) Serum ELISA analysis of NP-specific IgG1^b^ antibodies at 2, 4, and 8 week post-immunization with NP-CGG. Each data point represents one chimeric animal. No significant differences were observed by Mann-Whitney non-parametric test. (C) ELISA measurements of relative affinity of NP-specific serum antibodies. Data were obtained by quantifying the ratio of high affinity (NP_4_-binding) to total (NP_16_-binding) NP-specific antibodies at 4 and 8 weeks post-NP-CGG immunization. No significant differences were observed by Mann-Whitney non-parametric test. (D) Serum ELISA analysis of WNV envelope-specific antibody titers 8 weeks post-infection. Each data point represents *Gpr18* ^*+/+*^ or *Gpr18* ^*-/-*^ animal. Mean values ± SEM are shown. No significant differences were observed by Mann-Whitney non-parametric test. (E) Weight of *Gpr18* ^*+/+*^ and *Gpr18* ^*-/-*^ bone marrow chimeras following infection with influenza A virus. Mean values ± SEM are shown, n = 9 mice. No significant differences using 2-tailed students' unpaired t-test were observed. ALP, all-lymphoid progenitor; BLP, B lymphoid progenitor; A-E, Hardy fractions A, B, C, D, and E; FO, follicular B cell; TR, transitional B cell; SW, isotype switched B cell.

Analysis of the Immunological Genome Project dataset revealed that follicular B cells express GPR18 mRNA at similar levels as do intestinal IELs (Fig 1A). We confirmed high levels of GPR18 expression in follicular B cells through qRT-PCR analysis (Fig 8A). To determine if GPR18 has a B cell-intrinsic role in mediating antibody responses, we generated mixed bone marrow chimeras. Wild type IgH^a^ allotype bone marrow was mixed with equal numbers of IgH^b^ *Gpr18* ^*+/+*^ or *Gpr18* ^*-/-*^ bone marrow and transplanted into 800 cGy-irradiated CD45.1^+^ recipients. At 8 weeks post-transplant, animals were immunized with 4-hydroxy-3-nitrophenyl-acetyl (NP) conjugated to chicken gamma globulin (CGG), a T-dependent hapten-protein conjugate antigen. Allotype-specific secondary antibodies were then used to distinguish donor-derived antibodies by ELISA. NP-specific IgG1^b^ levels derived from *Gpr18* ^*+/+*^ and *Gpr18* ^*-/-*^ donors were equivalent at 2, 4, and 8 weeks post-immunization (Fig 8B). Thus, GPR18 deficiency does not affect antibody titers following immunization.

Although total antigen-specific antibody titers are normal in *Gpr18* ^*-/-*^ animals, these data do not exclude defects in affinity maturation or antibody quality. Such defects could in turn reflect problems in upstream germinal center reactions. Indeed, GPR18 shows a similar expression pattern as EBI2, a hydroxysterol receptor which is essential for B cell positioning in the germinal center reaction [79]. Affinity maturation of NP-specific antibodies can be estimated by quantifying binding ratios of antibodies to low (NP_4_) and high (NP_16_) densities of antigen. However, we found no defects in the affinity maturation of NP-specific antibodies derived from *Gpr18* ^*-/-*^ B cells (Fig 8C). These results demonstrate that GPR18 is not required to mount productive antibody responses.

The molecular requirements for mounting a productive antibody response to a hapten adjuvanted with aluminum salts may be different than to infections. For example, the transcription factor ZBTB20 is required for long-term antibody responses following immunization with alum-adjuvanted antigens, but is not required for other types of infections or vaccinations [80]. Thus, we infected *Gpr18* ^*-/-*^ mice with West Nile virus (WNV) and quantified antibody titers directed against the envelope protein. *Gpr18* ^*-/-*^ mice generated equivalent titers of WNV-specific antibodies as *Gpr18* ^*+/+*^ littermates (Fig 8D). These data suggest that despite its high level of expression in follicular B cells, GPR18 is not required for mounting antibody responses.

GPR18 is expressed at varying levels in many other cell types within the immune system (Fig 1A). Therefore, to further test the IEL-specificity of GPR18 function, we infected *Gpr18* ^*+/+*^ and *Gpr18* ^*-/-*^ bone marrow chimeras with influenza virus strain A/Puerto Rico/8/1934 H1N1. Mice with specific defects in innate or adaptive immunity lose more weight than do wild type mice at characteristic timepoints following influenza infection [81]. *Gpr18* ^*-/-*^ and control chimeras lost and regained weight after infection with equivalent kinetics (Fig 8E). Thus, GPR18 is dispensable for systemic immune responses and development, and is instead required specifically for the reconstitution of small intestine IELs.

## Discussion

Intestinal IELs play important roles in combating infections and in epithelial repair [53]. These IELs likely require the concerted action of several GPRs to position themselves and execute their functions, yet the identities of these receptors are not fully known [13]. Here, we have shown that GPR18 regulates the reconstitution of specific IEL subsets in the small intestine following bone marrow transplantation. Major deficits were not observed in the large intestine or under steady-state conditions. Moreover, despite high levels of expression in other cell types within the immune system, GPR18 is dispensable for hematopoietic development and responses to viral infections. Our data reveal highly selective requirements for GPR18 in IELs, dependent on both the subset of cells and their location.

Wang and colleagues recently reported similar observations regarding GPR18 in intestinal IELs [43]. Our data largely agree with and extend upon their findings, although one minor difference was noted. Wang et al. observed small defects in *Gpr18* ^*-/-*^ CD8αα^+^ TCRγδ^+^ IEL numbers in the steady-state. We did not observe defects under these conditions, however much of the other data are in concordance. Indeed, defects in all *Gpr18* ^*-/-*^ CD8αα^+^ subsets post-transplantation were observed in both studies. Our study also provides additional evidence of specificity, in that defects were not observed in large intestine IELs. We also find that GPR18 is dispensable for the thymic-independent pathway of IEL reconstitution. Moreover, despite the high levels of expression in follicular B cells, we demonstrate that GPR18 has no apparent role in lymphoid development or induction of antibody responses. Moreover, Wang and colleagues found no defects in the homing of mature T cells to the intestine [43]. Thus, GPR18 is selectively important for specific small intestine IEL subsets following transplantation.

One potential utility of identifying physiological agonists and synthetic antagonists of GPR18 is in the context of inflammatory intestinal diseases. Although IELs play important roles in the prevention of infection and epithelial repair, they can also contribute to disease pathogenesis [53]. A recent genome-wide association study identified a polymorphism near the *Gpr18* locus linked to inflammatory bowel disease [82]. This observation raises the possibility that the manipulation of GPR18 signals could be used to modulate inflammatory intestinal disorders.

GPRs generally serve as excellent and specific drug targets due to favorable structural characteristics. Indeed, nearly half of all small molecule drugs target GPRs [83]. Yet the physiological specificity of drugs also depends upon the breadth of *in vivo* functions of the target GPR. Here we have shown that, despite high levels of expression in other cell types, the function of GPR18 is remarkably specific for small intestinal IELs. Our data raise the possibility that manipulation of GPR18 might modulate IEL behavior selectively in specific contexts of health and disease.

## Supporting Information

S1 Fig Flow cytometric gating strategy for steady state and post-transplant donor-derived IELs. Small intestinal IELs were isolated, and TCRαβ and TCRγδ cells were identified as shown. Expression of CD8α and CD8β was used to subset the populations as shown, and the percentage of CD45.2^+^ donor-derived cells was quantified by gating as shown.
(TIFF)

S2 Fig Flow cytometric gating strategy for hematopoietic progenitors. Viable lineage^-^ (lacking expression of B220, CD3ε, Ter119, CD11b, and Ly6C/G) bone marrow cells were stained and gated as shown. HSC, hematopoietic stem cell; MPP, multipotent progenitor; ALP, all-lymphoid progenitor; BLP, B lymphoid progenitor; CMP, common myeloid progenitor; GMP, granulocyte macrophage progenitor; MDP, monocyte dendritic cell progenitor; CDP, common dendritic cell progenitor.
(TIFF)

S3 Fig Flow cytometric gating strategy for mature splenic lineages and B cell progenitors. (A) Splenocytes were gated as shown to identify B cells, T cells, monocytes, and granulocytes. (B) Bone marrow cells were stained for Hardy Fractions A-E as shown. (C) Strategy to identify mature B cell subsets in the spleen.
(TIFF)

S1 Table Flow cytometric antibodies. Antibody specificities, clone numbers, fluorescent conjugates, and vendors and sources are shown.
(TIF)
