# Supplementary figures and images for: GPR18 Controls Reconstitution of Mouse Small Intestine Intraepithelial Lymphocytes following Bone Marrow Transplantation
Source: PLoS One. 2015 Jul 21;10(7):e0133854. doi: 10.1371/journal.pone.0133854 (PMC4510063; doi:10.1371/journal.pone.0133854)

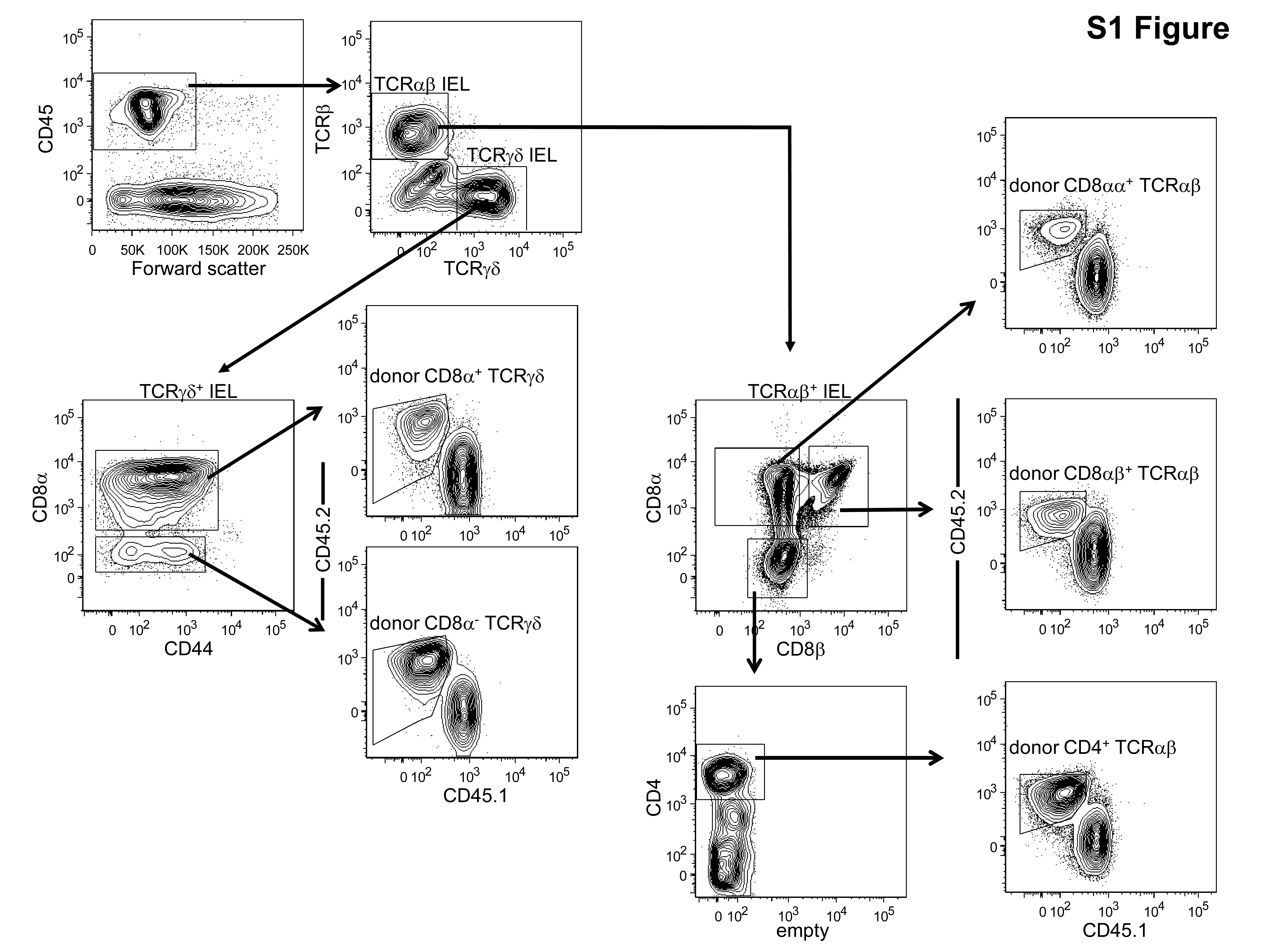

Supplement: S1 Fig — Small intestinal IELs were isolated, and TCRαβ and TCRγδ cells were identified as shown. Expression of CD8α and CD8β was used to subset the populations as shown, and the percentage of CD45.2+ donor-derived cells was quantified by gating as shown. (TIFF) [file pone.0133854.s001.tiff]

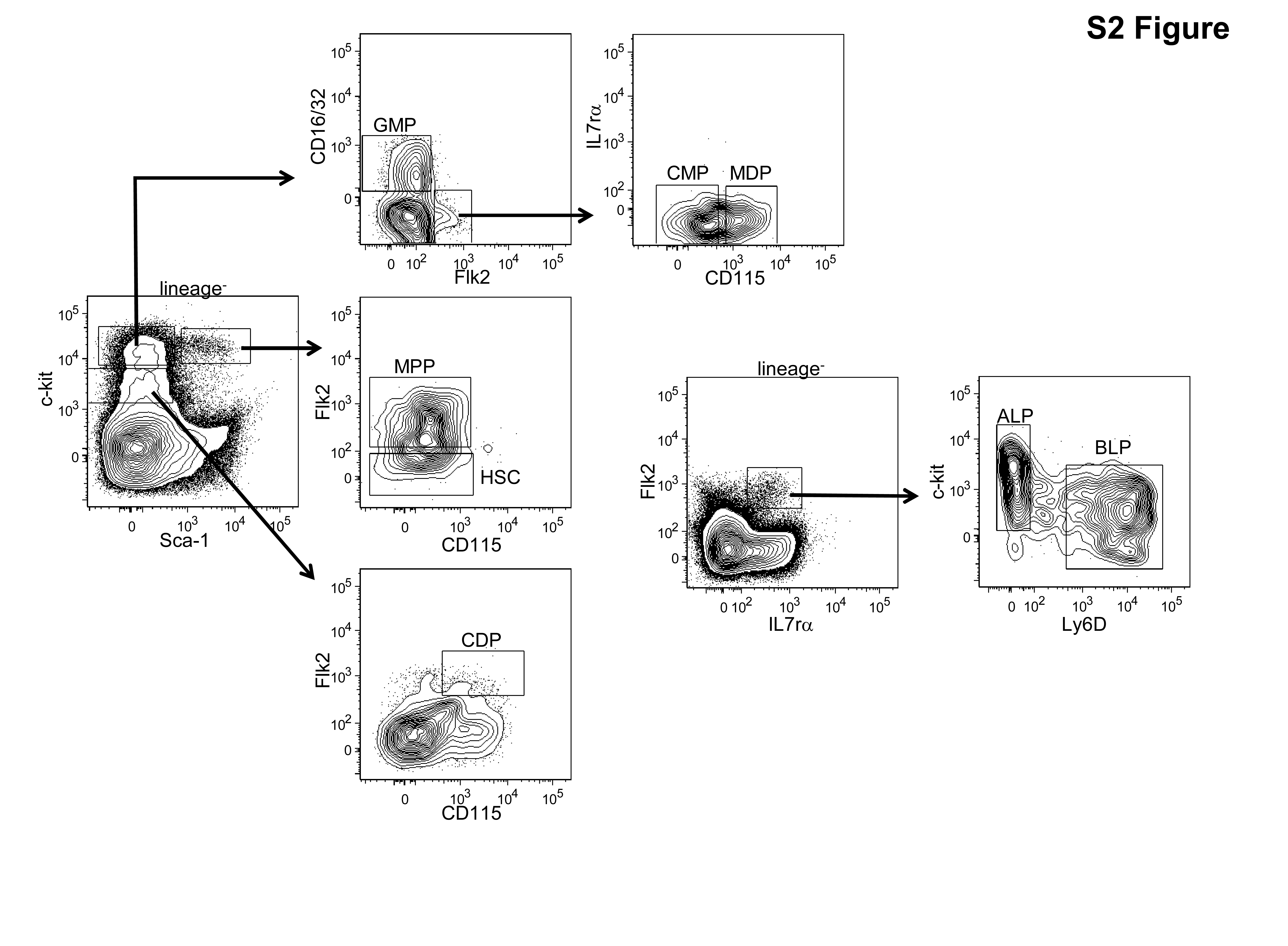

Supplement: S2 Fig — Viable lineage- (lacking expression of B220, CD3ε, Ter119, CD11b, and Ly6C/G) bone marrow cells were stained and gated as shown. HSC, hematopoietic stem cell; MPP, multipotent progenitor; ALP, all-lymphoid progenitor; BLP, B lymphoid progenitor; CMP, common myeloid progenitor; GMP, granulocyte macrophage progenitor; MDP, monocyte dendritic cell progenitor; CDP, common dendritic cell progenitor. (TIFF) [file pone.0133854.s002.tiff]

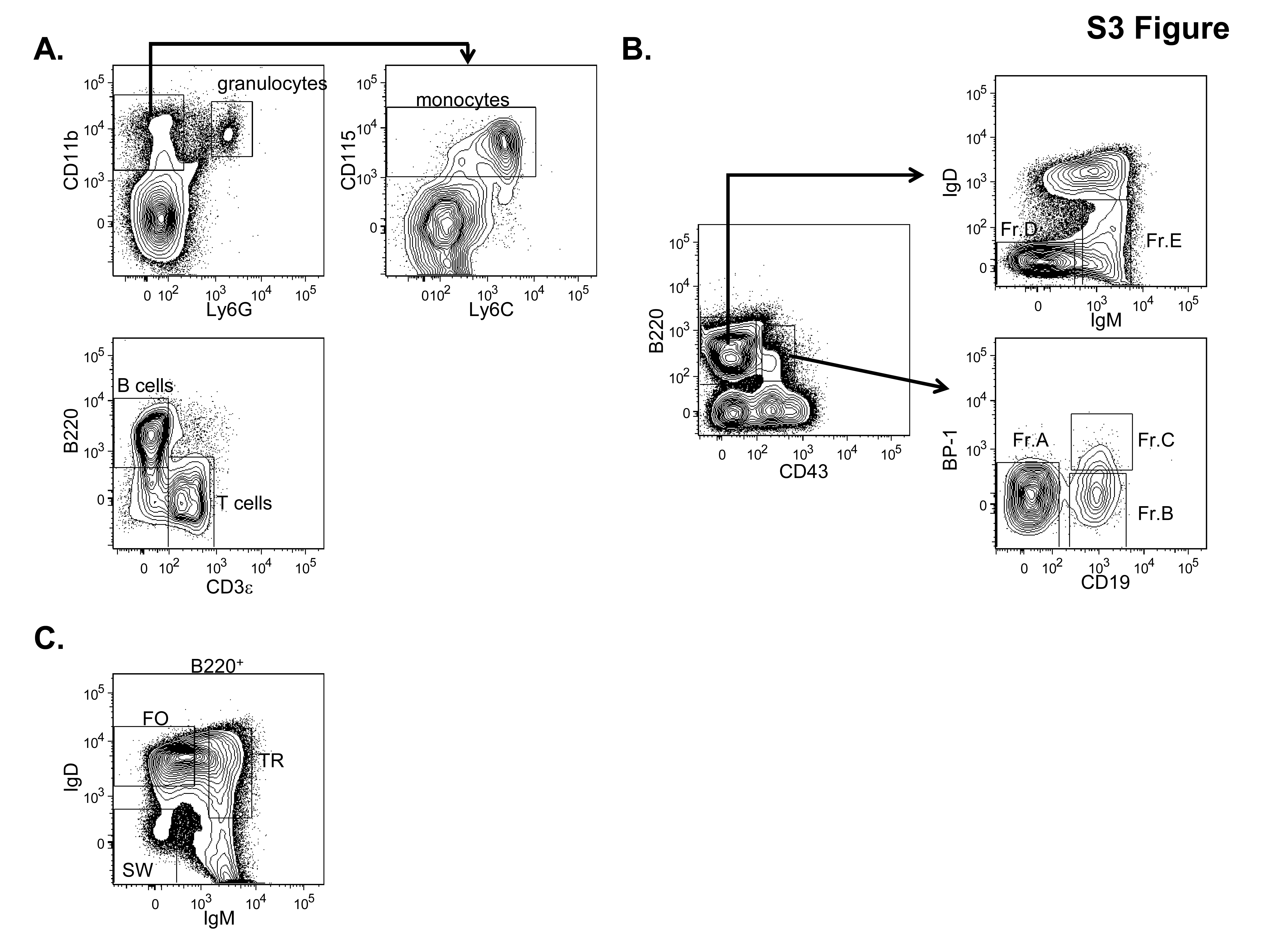

Supplement: S3 Fig — (A) Splenocytes were gated as shown to identify B cells, T cells, monocytes, and granulocytes. (B) Bone marrow cells were stained for Hardy Fractions A-E as shown. (C) Strategy to identify mature B cell subsets in the spleen. (TIFF) [file pone.0133854.s003.tiff]

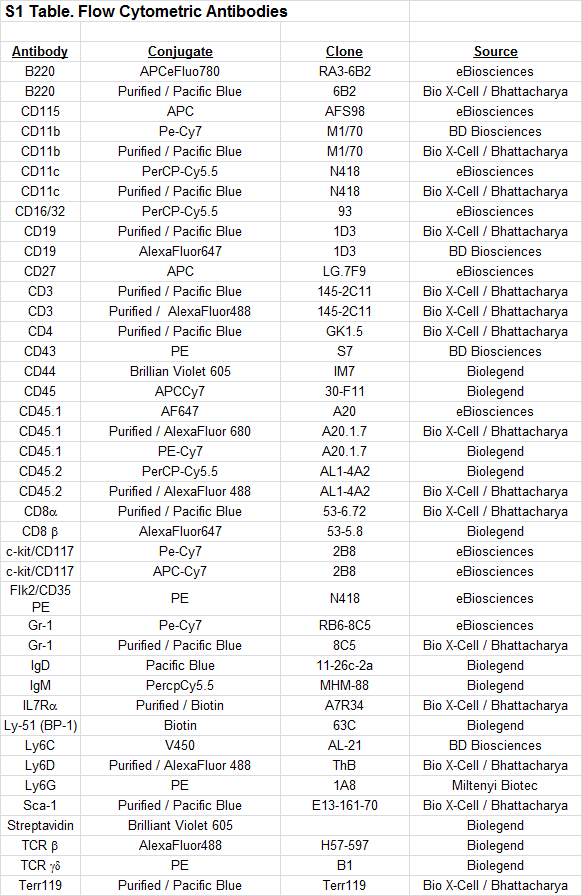

Supplement: S1 Table — Antibody specificities, clone numbers, fluorescent conjugates, and vendors and sources are shown. (TIF) [file pone.0133854.s004.tif]
